# Validated UHPLC Methods for Melatonin Quantification Reveal Regulatory Violations in EU Online Dietary Supplements Commerce

**DOI:** 10.3390/molecules30122647

**Published:** 2025-06-19

**Authors:** Celine Vanhee, Cloë Degrève, Niels Boschmans, Yasmina Naïmi, Michael Canfyn, Eric Deconinck, Marie Willocx

**Affiliations:** Service Medicines and Health Products, Scientific Direction of Chemical and Physical Health Risks, Sciensano, J. Wytsmanstraat 14, B-1050 Brussels, Belgium

**Keywords:** chromatography, method validation, mass spectrometry, analytical chemistry, dietary supplements, quality control, public health

## Abstract

The global sleep aids market has grown significantly due to rising stress and increased awareness of sleep’s importance. Melatonin, available in the EU as a prescription or over-the-counter medicine, depending on the country, is also permitted in dietary supplements with country-specific limits. Recent reports indicate concerning levels of excessive melatonin in EU dietary supplements, necessitating accurate quantification methods. We developed and validated, by applying accuracy profiles, ISO17025-compliant, rapid ultra-high performance liquid chromatography (UHPLC) methodologies coupled with either diode array detection (DAD) or high-resolution accurate mass spectrometry (HRAM MS). The cost-effective UHPLC-DAD method is suitable for medicines and most dietary supplements, except those more complex herbal matrices containing passionflower, hop, hemp, lime tree or lavender or their extracts, where UHPLC-HRAM MS is recommended due to selectivity issues of the DAD methodology. To demonstrate the applicability, we analyzed 50 dietary supplements claiming to contain melatonin—25 from legal supply chains and 25 from suspicious sources claiming therapeutic melatonin content. Our findings confirmed previous reports of high melatonin content in online products, especially when purchased through rogue internet pharmacies. Moreover, 12% of legal supply chain samples violated current legislation through unauthorized health claims or contained at least triple the melatonin amount permitted in Belgium. This research provides reliable analytical methods for regulatory bodies and confirms the circulation of non-compliant melatonin-containing dietary supplements in the EU market, even in the legal supply chain.

## 1. Introduction

It is estimated that we spend about one-third of our lives either sleeping or attempting to do so. Poor or insufficient sleep can affect one’s professional performance and/or mental well-being, while if occurring chronically, it can also increase the likelihood of developing several detrimental health conditions. Many reports, originating from the United States of America (USA), the United Kingdom (UK), and several countries in the European Union (EU), indicate an increase in the usage of exogenous melatonin to treat sleep disorders in both adults and children [1,2,3,4,5,6,7]. Moreover, it is also used temporarily to aid with jet lag.

Melatonin (N-acetyl-5-methoxytryptamine) is an indoleamine, mainly produced by the pineal gland, and its biosynthesis and release into the bloodstream and cerebrospinal fluid are stimulated as a response to darkness. From there on, it can reach several areas of the central nervous system (CNS) and all peripheral organs, where it will trigger different effects by various mechanisms of action [8], including sleepiness induction and circadian rhythm regulation in humans. Generally, exogenous melatonin intake at concentrations of 0.3 to 10 mg daily may help increase total sleep time in adults with sleep restriction or altered sleep schedules; relieve daytime fatigue associated with jet lag; reset the sleep-wake cycle; and reduce time to fall asleep in people with delayed sleep phase syndrome [9,10,11,12]. Currently, there is no official maximum dosage set for adults and no consensus regarding dosage. However, a wide range of dose formulations are available, and the usage varies depending on the clinical application [13]. Low doses from 0.1 to 0.3 mg per day are thought to produce near-physiological melatonin concentrations and can be used for central clock synchronization. Doses ranging from 0.6 to 10 mg per day are often used for sleep disorders. Although the compound is considered generally safe for short-term use by adults, therapeutic dosages exceeding 10 mg per day are not recommended as this could increase the possibility of unwanted side effects [14]. The long-term effects of melatonin supplementation remain uncertain. In contrast to adults, lethal melatonin overdoses have been reported in children [15]. According to the Centers for Disease Control (CDC), unsupervised melatonin ingestion was implicated in approximately 11,000 (7%) emergency department visits among infants and young children during 2019–2022. Many incidents involved the ingestion of flavored products (e.g., gummy formulations) [16]. In the USA, melatonin is regulated as a dietary supplement, while in Australia, the United Kingdom (UK), and Switzerland, it is regulated as a prescription medicine; consequently, its presence is not allowed in dietary supplements [17,18,19]. For the members of the European Union (EU), the specific regulation depends on the country (see Table 1). In general, it can be said that, except for Poland, prolonged-release melatonin is considered a prescription medicine while immediate-release melatonin, if available, is either a prescription medicine or an over-the-counter (OTC) medicine [20]. In some EU countries, melatonin is allowed up to a certain concentration in dietary supplements (see Table 1). In France, the country with the highest concentration of melatonin permitted, dietary supplements can contain a maximum of 2 mg per daily dose [21]. However, in a recent market surveillance study performed by 11 European Official Medicines Control Laboratories (OMCL) and the Australian Therapeutic Goods Administration (TGA), several samples were described that contained high quantities of melatonin (>2 mg per daily intake) and some even exceeded the recommended therapeutic daily limit of 10 mg [22]. Therefore, it is essential for controlling agencies to accurately quantify this molecule in a variety of dietary supplements to verify their compliance with national legislation or to perform a market surveillance study to see what is available to the public—certainly, bearing in mind the increasing popularity of these supplements. Although several quantification methodologies have been generated in the past, often employing high-pressure liquid chromatography (HPLC), to our knowledge, none of them have been validated according to the total error approach or the fitness for purpose approach, compliant with both ICH and ISO17025 [3,23,24,25,26,27,28,29]. Through this approach, it is possible to verify if a method’s overall error is acceptable for its intended use [30]. The latter is essential if further legal steps are anticipated. In this paper, we describe the generation and the validation of a simple and fast ultra-high-performance liquid chromatography (UHPLC) coupled to either a DAD, applicable to both medicines and many different dietary supplements. Moreover, the newly developed UHPLC methodology could also be coupled to high-resolution accurate mass (HRAM) mass spectrometry (UHPLC-HRAM MS). This approach, in combination with the use of stable isotope-labeled melatonin, was developed for the quantification of low-dose melatonin in more complex herbal dietary supplements containing *Passiflora incarnata*, *Humulus lupulus*, *Cannabis sativa*, *Tilia* sp., and lavender or extracts thereof as overlapping peaks were observed by DAD detection. Both quantification methodologies were validated using the “total error approach”, by applying accuracy profiles. Next, 50 real-life supplements purchased from either the legal or suspect supply chain were analyzed.

## 2. Results and Discussion

### 2.1. Development and Validation of the UHPLC-DAD Quantification Method

The chromatographic separation was performed at 30 °C on an Acquity™ UPLC CSH column (100 × 2.1 mm, 1.7 μm particle size). This column was chosen as it provided the most reproducible results for challenging matrices, compared to a classical BEH C18 column [31]. Solvent A (0.1% formic acid in water) and solvent B (methanol in the case of DAD detection or methanol supplemented with 0.1% formic acid in the case of detection by MS) were compatible with MS-based detection techniques. The total run time equaled 4 min and resulted in the consumption of only 1.2 mL liquid, including 480 µL methanol. For the detection of target analytes, detection through a DAD was initially favored, as in contrast to MS-based technologies, it does not require the use of an expensive stable isotope-labeled reference standard. This detection method is particularly valuable considering that melatonin in solution is not stable for long periods, even when stored at −20 °C in the dark [32,33,34].

The UHPLC-DAD separation method was subsequently validated by assessing the selectivity and specificity. This was achieved by checking the absence of a peak with a similar retention time as the pure melatonin reference standard in different matrices. Melatonin peak identification was confirmed by comparing both retention time and ultraviolet spectral characteristics of a reference standard with those of melatonin-fortified matrices. For all samples, we verified that the purity angle was below the purity threshold, confirming the chromatographic peak’s integrity and absence of co-eluting compounds. The tested matrices were chosen based on the other ingredients listed for the different supplements (see Supplementary Materials Table S1) and consisted of (1) dried *Valeriana officinalis* powder; (2) a dietary supplement containing different vitamins B and vitamin C; (3) *dried Matricaria chamomilla* (Camomille) powder; (4) dried *Eschscholzia californica* (California poppy) powder; (5) dried *Melissa officinalis* powder; (6) dried *Papaver rhoeas* powder; (7) dried *Humulus lupulus* (hop) powder; (8) dried *Tillia* sp. (lemon tree) powder; (9) dried Cannabis sativa (hemp) powder; (10) dried *Lavendula angustifolia* (lavender) powder; and (11) dried *Passiflora incarnata* (passionflower) powder. As can be seen in Supplementary Materials Figure S1, no peak around 2.5 min could be observed for the first 6 matrices, while matrices 7 to 11 did show the presence of a peak eluting around this retention time. The biggest peaks were found in the extracts of hop and passionflower and differed from melatonin based on their UV spectrum (Supplementary Materials Figure S1). Based on the lack of selectivity in these five matrices, it was decided that the methodology did not apply to samples that claimed the presence of hop, passionflower, lime tree, hemp or lavender. However, the UHPLC-DAD methodology could still be used for those supplements that did not contain the abovementioned plant material, as this approach is economically favorable.

Subsequently, the lower limit of quantification (LLOQ) and the upper limit of quantification (ULOQ) were determined. The LLOQ was defined as the lowest concentration with a signal-to-noise ratio greater than 10 and the highest concentration for which accuracy and precision could be demonstrated, and corresponded to 5 µg/mL melatonin or 100 µg melatonin per gram sample (see Table 2). The ULOQ was determined as the highest concentration for which accuracy and precision could be demonstrated and corresponded to 250 µg/mL. Compared to previously published UHPLC-DAD methodologies, the obtained LLOQ is 10 times higher; however, these methodologies defined their LOQ as ≥10 signal-to-noise but did not apply any accuracy requirements for their LOQ [3,28].

Additionally, linearity was assessed for concentrations ranging from 5 to 250 µg/mL by applying least squares regression analysis (see Table 2). Adequate linearity was achieved with regression coefficients (r) ≥ 0.99. Moreover, the potential matrix effects were also evaluated by performing a *t*-test on the slope of the calibration curves for melatonin in those different matrices and by comparing the recovery of melatonin in 3 different matrices at five different concentration levels. The acceptance limits were set to 100% ± 20%. Both the *t*-test and the obtained recoveries (ranging from 97.6–104.8%), indicated the absence of significant matrix effects (see Supplementary Materials Table S2).

Next, the total error approach was used to determine the trueness, precision, and accuracy (see Table 3). The β-expectation tolerance limits, computed for each concentration level, serve as a predictive tool ensuring that 95% of future analytical results will remain within the predefined acceptance range of [−5; 5], a criterion commonly applied in pharmaceutical preparations.

**Figure 1:**
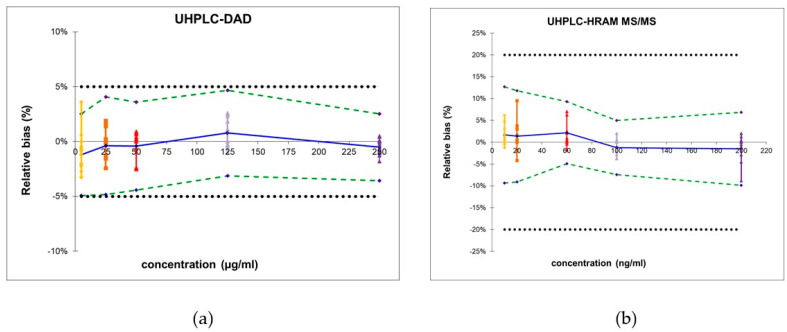
Accuracy profiles of melatonin when quantified by UHPLC-DAD (**a**) or UHPLC-HRAM MS (**b**) with the β-expectation interval (black dotted line), relative bias (solid blue line), 95% β-expectation tolerance limits (dashed green line); back-calculated concentrations of reference concentrations at the LLOQ (yellow), ULOQ (purple), and three intermediate concentrations (orange, red, and lilac).

As visualized in Figure 1a, the obtained β-expectation tolerance limits did not exceed the acceptance limits, rendering the method suitable for the quantification of pharmaceuticals and several different dietary supplements, provided hop, passionflower, lime tree, hemp or lavender are not present in the sample.

### 2.2. Development and Validation of the UHPLC-HRAM MS Quantification Method

The newly developed targeted MS methodology followed the same validation strategy as abovementioned UHPLC-DAD approach. The method was shown to be selective and specific as no interference could be observed from all the tested matrices.

The LLOQ and ULOQ corresponded to 10 and 200 ng/mL, respectively, and demonstrated a linear response within this concentration range (see Table 2). Similar to the UHPLC-DAD methodology, our obtained LLOQ is higher than the previously reported LLOQ [29]. However, the previously reported methodologies had a different definition, as we also included the accuracy requirement for the LLOQ.

Moreover, no significant matrix effects could be observed as the recoveries in different matrices ranged from 92.7 to 110.3% (see Supplementary Materials Table S3). Next, the trueness, precision, and accuracy were determined (see Table 3). Moreover, the obtained β-expectation tolerance limits did not exceed the predefined acceptance limits of ±20% (see Figure 1b). These limits do not apply to pharmaceutical preparations containing melatonin; however, the developed methodology can be used to quantify melatonin in dietary supplements containing substances that interfere with the UHPLC-DAD methodology.

### 2.3. Analysis of Real-Life Samples

#### 2.3.1. Ease of Purchase, Labeling, and Presence of Child-Proof Lid for Gummies

All samples arrived within one week of purchase. The legal melatonin samples were sent from Belgium, Italy, or Germany. The Italian and German samples were obtained through a Belgian online pharmacy registered with the Federal Agency for Medicines and Health Products (FAMHP). The Belgian samples displayed information in both Dutch and French (the main languages in Belgium) and labeled the presence of daily amounts less than 0.3 mg. The Italian and German samples (L18 and L25), on the other hand, mentioned the presence of 1 mg and 3 mg of melatonin, respectively (see Table 4). Although purchased from a registered online pharmacy, the Italian and German products do not comply with Belgian legislation as they exceed the daily intake limits that are set at 0.3 mg. According to Belgian law, these samples should be subject to regulatory scrutiny. However, the 1 mg daily dose complies with Italian regulations, where the product originated. The German sample, on the other hand, might or might not comply with German legislation, as currently the situation for melatonin in dietary supplements is not clear in Germany and depends on the court. Nevertheless, this phenomenon could be the result of overlooked differences in national regulations (see Table 1). It stands to reason that consumers, manufacturers and distributors would benefit from a more homogeneous regulation for melatonin-containing dietary supplements amongst the different EU member states or associated trade countries (see Table 1).

Prior to chemical analysis, we also screened the dietary supplement labels for batch numbers and expiration dates, which are mandatory according to European legislation [35,36]. As shown in Table 4, only one sample from the suspicious supply chain lacked an expiration date. Next, sample labeling was also evaluated according to European Food Safety Authority (EFSA) guidelines, which require at least 1 mg of melatonin before bedtime to substantiate sleep aid claims [37]. Therefore, supplements containing less than 1 mg per daily dose cannot legally claim sleep aid benefits based solely on melatonin content. Indeed, most samples from the legal supply chain attributed sleep-related health claims to ingredients other than melatonin. However, three of the 25 samples (L3, L18 and L25) mentioned sleep aid claims without attributing them to alternative ingredients. For the German sample, these claims are in agreement with EFSA regulation as it contained more than 1 mg melatonin (see Table 4). Additionally, we examined 25 samples purchased specifically for their high melatonin content. These were shipped from either The Netherlands or Bulgaria, and almost all of these samples claimed to be manufactured in the USA (22 out of 25). The three other samples claimed to be manufactured in Romania (I21 and I22) or India (I25). Unlike the samples from the legal supply chain, all 25 of these samples included health claims directly related to melatonin presence.

Although EFSA does not require warnings on labels for melatonin-containing dietary supplements, we compared the presence of warnings and contraindications across products. As detailed in Supplementary Materials Table S1, legal supply chain samples exhibited substantial variation in warnings, ranging from simple statements like “keep out of reach of children” to elaborate contraindications concerning age, pregnancy, lactation, and various health conditions that would necessitate consulting healthcare professionals before use. A more standardized approach to warnings would benefit both consumers and manufacturers. In contrast, samples from the suspicious supply chain consistently included contraindications related to age, pregnancy, lactation, and various health issues.

Moreover, as gummies are often the source of accidental melatonin overdose in children, it was also investigated if these products and oral strips contained a child-proof cover. The latter has been proposed by the Council for Responsible Nutrition following the many pediatric melatonin intoxications that occurred in the USA in recent years [38]. Only one of the two gummy samples from the illegal supply chain contained such a cover, while a child-proof lid was present in all gummy samples originating from the legal supply chain.

#### 2.3.2. Determination of the Melatonin Content

Before quantification, the presence of melatonin and other non-listed active ingredients was first verified by our routine LC-MSn screening methodology, used in our official medicines laboratory (OMCL) to screen suspected illegal medicines or medicines in disguise [39,40,41]. The screening detection limit (SDL) for melatonin corresponded to 0.5 µg/mL. Upon analysis by this broad screening approach, all samples were positive for the presence of melatonin (LOD for melatonin of this screening methodology), and no other active ingredient other than the ones listed were detected.

**Figure 2:**
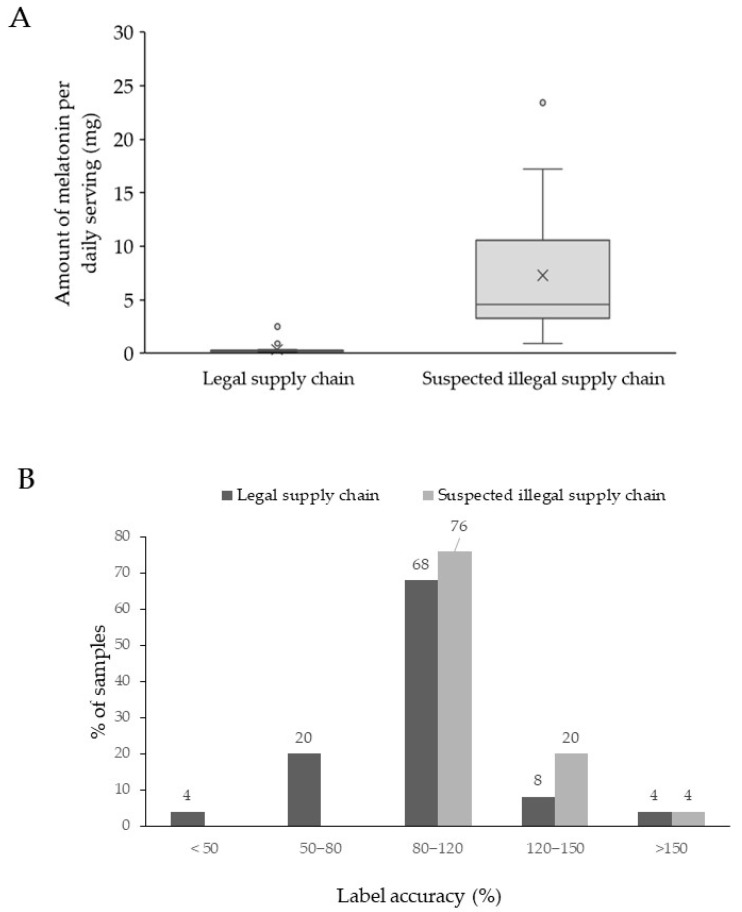
Box and whisker plot for the samples originating from the legal and suspected illegal supply chain (**A**) and the percentage label accuracy found for the samples coming from the different supply chains (**B**). For the box plots, the bottom and top of the box correspond to the lower quartile (25th percentile) and the upper quartile (75th percentile), respectively. The line inside the box represents the median (50th percentile), while the X represents the mean value. The ends of the whiskers correspond to the lowest and highest observations that are still within 1.5-fold of the interquartile range (corresponding to the length of the box).

Next, the amount of melatonin was quantified using UHPLC-DAD for all illegal samples, except for those samples containing passionflower (I20 and I25). A quantification with the DAD was also performed for 12 of the samples of the legal market that did not list any herbal extract other than *Valerian* sp. or vitamins. The remaining 13 samples were analyzed by LC-HRAM MS, as potential matrix interference could not be excluded, certainly taking into account the low amount of melatonin that is listed on the label. The outcome from this quantification is summarized in Table 4. There, it can be found that the label accuracy of the samples originating from the legal supply chain ranged between 71–182% label accuracy. Amounts ranged from 0.11 to 2.5 mg, with an average of 0.4 mg and a median of 0.24 mg. Only three samples from the legal supply chain exceeded the maximum daily dosage of 0.3 mg by 120%; however, according to the box and whisker plot (Figure 2A), only the Italian (L18) and German (L25) samples were considered outliers. They contained 0.9 and 2.5 mg of melatonin per serving, respectively. Also, sample L23 exceeded this 120% limit and contained 0.38 mg per serving, corresponding to 129% of the labeled amount.

Although EFSA does not define the tolerance limits for melatonin in dietary supplements, a −20% to a +50% deviation is allowed for vitamins in dietary supplements [42]. When extrapolating these EFSA regulations for vitamins to the melatonin samples obtained through the legal supply chain, only 6 samples are not compliant, as one sample contained 182% of the labeled amount (L7), while the remaining six samples contained less than 80% (see Figure 2B). These five samples (L1, L5, L10, L17, and L24) contained between 70 and 80% of the labeled amount, while sample L8 contained only 40% of the labeled amount. In summary, it can be stated that two of the samples obtained from the legal supply chain are forbidden in Belgium and that 24% of the authorized samples contained less than 80% of the labeled content. Although this does not represent a danger to public health, it could serve as an incentive to establish tolerance limits for label accuracy, certainly regarding compounds with restricted dosage allowances in food supplements.

All samples from the suspicious supply chain contained melatonin exceeding 0.3 mg per daily dosage. Amounts ranged from 0.91 to 23.43 mg, with an average of 7.3 mg and a median of 4.58 mg (see Figure 2A). Next, an ANOVA was performed on the quantification and demonstrated a significant difference in the amount of melatonin found in samples from the legal and suspected illegal supply chains (*p*-value < 0.001). Moreover, in contrast to the legal supply chain, all studied samples contained at least 80% of the labeled amount, as the label accuracy varied between 82% and 172% (see Figure 2B). These findings resemble the data obtained from previous USA market surveillance studies [3,29]. However, these studies also reported the occurrence of samples that contained more than 300%, even up to 600% of the labeled amount, but this occurred only in very few samples. No such label deviation was observed in our sample set originating from the USA. However, four of the 25 samples (16%) exceeded the recommended therapeutic daily limit of 10 mg per day by at least 120%. Such high doses increase the risk of adverse effects, including drowsiness, dizziness, fatigue, headache, confusion, nightmares, hypotension, tachycardia, hypothermia, and exacerbation of pre-existing mental conditions. These findings corroborate previous reports that supplements containing therapeutic amounts of melatonin are circulating in the EU market and are readily available online [22]. For consumer protection, these high-melatonin samples should be subject to regulatory scrutiny, and websites promoting or selling these items from within Europe should be banned.

Taken together, this study confirms previous reports of high melatonin content circulating within Europe. Our results show these items frequently come from the USA, where melatonin is not regulated and where studies have documented inconsistencies between labeled and actual melatonin content in supplements, raising potential safety concerns, especially for children. Moreover, our study demonstrates that at least 3 samples (12%) of legal supply chain samples violated current legislation through unauthorized health claims (L6 and L25) or contained at least triple the melatonin amount permitted (L18 and L25) in Belgium. Two non-compliant samples were purchased through a registered online pharmacy and are likely circulating in the Belgian market due to regulatory gaps. These products, while legal in their countries of origin, fail to meet Belgium’s specific standards, highlighting a critical regulatory disconnect across EU member states. Moreover, these findings underscore the urgent need for more accessible, transparent, and harmonized regulation of dietary supplements throughout the European Union. The varying supplement regulations across EU countries create confusion in today’s digital marketplace, where consumers frequently purchase products from other member states without realizing they may not comply with their own country’s standards. Moreover, the current information on regulatory status often requires consumers to navigate complex national regulations—a process very few undertake when seeking products to improve health or address specific conditions. As the sleep aids market continues its robust growth, establishing clearer and more uniform standards for supplements becomes increasingly critical for public health and market integrity. Additionally, the establishment of tolerance limits for label accuracy by EFSA would prove beneficial to analytical laboratories and regulatory agencies, particularly for compounds subject to quantity restrictions in food supplement formulations.

## 3. Materials and Methods

### 3.1. Sample Set

A total of 25 dietary supplements branded to contain melatonin were purchased online from online pharmacies licensed by Belgian health authorities or from websites of some well-known brick-and-mortar drug stores. Another 25 samples were purchased online from EU-based e-shops claiming to sell products that contained more than 1 mg of melatonin per daily dose (search terms: melatonin, mg, buy, Europe, supplement). All online ordered samples arrived within one week of ordering and were stored in the dark at room temperature (15 to 25 °C) before analysis. The analysis took place before the expiration date.

### 3.2. Quantification of Melatonin

#### 3.2.1. Solvents, Reagents, and Standard Solutions

Mass spectrometry (MS)-grade methanol (purity > 99.9%) and water were purchased from Thermo Fisher Scientific (Waltham, MA, USA), while MS-graded formic acid (purity > 99%) was purchased from Biosolve (Valkenswaard, The Netherlands). The reference standard for melatonin (purity 99.5%) was purchased from Fagron (Waregem, Belgium), while the melatonin-d3 (purity 99.8%) was purchased from MedChemTronica (Sollentuna, Sweden). Standard stock solutions of 5 mg/mL of both melatonin and melatonin-d3 were prepared in methanol, kept in the dark at −20 °C, and used within fourteen days.

To determine the limit of quantification, serial dilutions were made from these standard stock solutions in methanol-water (50:50) *v*/*v*. These working solutions were used within 24 h with minimum light exposure. At least 6 different concentrations were used for the generation of the calibration curves. Next, to check the extent of the effects of the matrix, the working solutions were diluted in methanol-water (50:50) *v/v* extracts of the chosen matrices. The matrix extracts were made by extracting 100 mg of dry matrix powder in 10 mL methanol-water (50:50) *v/v*. Based on the other ingredients often also present in the sample set (see Supplementary Materials Table S1), the following items were tested for interference: (1) dried *Valeriana officinalis* powder, (2) a dietary supplement containing different vitamins B and vitamin C, (3) dried *Matricaria chamomilla* powder, (4) dried *Eschscholzia californica* powder, (5) dried *Melissa officinalis* powder, (6) dried *Papaver rhoeas* powder, (7) dried *Humulus lupulus* powder, (8) dried *Tillia* sp. (lime tree) powder, (9) dried Cannabis sativa powder, (10) dried *Lavendula angustifolia* powder and (11) dried *Passiflora incarnata* powder. The reference material for the different plant species was either bought from Fagron (Waregem, Belgium), collected in the wild and dried, and pulverized in the lab, or was bought as a dietary supplement claiming to be devoid of melatonin but containing different vitamins often also present in dietary supplements containing melatonin (see Supplementary Materials Table S1).

#### 3.2.2. Instrumental Settings UHPLC-DAD

The amount of melatonin in dietary supplements was determined on a Waters Acquity UPLC™ H-class system (Waters Corp., Milford, MA, USA) including a quaternary solvent manager, sample manager-flow through needle, column heater, and diode array (DAD) detector connected to Waters Empower 3.7.0 data station. The chromatographic separation was performed at 30 °C on an Acquity™ UPLC CSH Column (100 × 2.1 mm, 1.7 μm particle size) (Waters, Milford, MA, USA) with a mobile phase consisting of 0.1% formic acid in water (A) and methanol (B) at a flow rate of 0.3 mL/minute. The developed elution method employed an isocratic run for 5 min at 40% B. Melatonin was monitored at a wavelength of 278 nm.

#### 3.2.3. Instrumental Settings UHPLC-HRAM MS

High-resolution accurate mass (HRAM) tandem mass spectrometry was performed using a Thermo Scientific™ Vanquish™ ultra-high performance liquid chromatography system connected to a Q Exactive™ Focus orbitrap mass spectrometer (Thermo Fisher Scientific, Bremen, Germany).

The liquid chromatography conditions remained identical to those previously described, with the modification that mobile phase B contained 0.1% formic acid in methanol for optimal ionization. Mass spectrometric analysis utilized the Q-Exactive Focus Orbitrap instrument equipped with a heated electrospray ionization (HESI) source configured for positive ion detection. Operating parameters were optimized as follows: nitrogen sheath gas (≥99.99% purity) at 30 arbitrary units, nitrogen auxiliary gas (≥99.99% purity) at 10 arbitrary units, capillary temperature of 230 °C, spray voltage of 3 kV, and S-lens RF level of 50 V.

Full-scan mass spectra were acquired across *m*/*z* 100–300 with 70,000 resolving power (at *m*/*z* 200). Targeted analysis employed parallel reaction monitoring (PRM) at 17,500 resolving power, with collision-induced dissociation energy set to 30 eV for precursor ion fragmentation. The inclusion list monitored melatonin (*m*/*z* 233.128 [M + H]^+^) and the deuterated internal standard melatonin-d3 (*m*/*z* 236.147), as detailed in Table 2. Data acquisition utilized Thermo Xcalibur 4.4 software, while data processing was conducted with Tracefinder 5.1 software (Thermo Fisher Scientific, Bremen, Germany). High-resolution accurate mass (HRAM) tandem MS analyses were carried out on a Thermo Scientific™ Vanquish™ ultra-high performance liquid chromatography (UHPLC) system coupled to a Q Exactive™ Focus orbitrap mass spectrometer (Thermo Fisher Scientific, Bremen, Germany).

#### 3.2.4. Validation of the Quantification Methodology

Method validation for melatonin quantification followed ICH guidelines [43] and employed the total error approach [30] in accordance with ISO17025 standards [44], consistent with our research group’s previous validation work on food supplement analysis [39,40,41].

The limit of quantification (LOQ) was established as the lowest concentration demonstrating acceptable accuracy and precision, requiring a signal-to-noise ratio ≥10 and serving as the lowest calibration point for which accuracy and precision could be demonstrated.

Linearity assessment and matrix interference evaluation: Method validation included linearity testing of both DAD and PRM responses across concentration ranges of 5–250 µg/mL and 10–200 ng/mL, respectively, using least squares regression analysis with a minimum of six intermediate concentrations. Acceptable linearity required a correlation coefficient (r) ≥ 0.99. Mandel’s fitting test evaluated potential non-linearity by comparing quadratic versus linear regression models [45]. Matrix effects were assessed through *t*-test comparison of calibration curve slopes across different matrices, with all measurements performed in triplicate. Additional matrix effect evaluation involved recovery studies of melatonin in three distinct matrices at five concentration levels, with acceptance criteria of 100% ± 20%.

Method performance characteristics: The total error approach was implemented to evaluate method performance by combining systematic error (trueness) and random error (intermediate precision), enabling calculation of trueness, accuracy, precision, and estimation of total error and measurement uncertainty. β-expectation tolerance limits calculated at each concentration level provide predictive capability, ensuring 95% of future analytical results fall within predetermined acceptance limits [−λ; λ]. Acceptance boundaries were established at ±5% for UHPLC-DAD quantification and ±20% for UHPLC-HRAM MS quantification, with the wider tolerance reflecting the analytical challenges associated with low melatonin concentrations and complex herbal matrices when using MS detection.

Validation studies utilized daily preparation of spiked samples in triplicate across five concentrations spanning the lower and upper limits of quantification, analyzed over a minimum of three consecutive days. Concentrations were back-calculated using same-day calibration curves, and the resulting data were processed using a validated Excel template previously employed by our research group [39,40,41] to determine linearity, trueness, precision (both repeatability and intermediate precision), and accuracy.

### 3.3. Sample Preparation and Market Study

Most samples consisted of tablets or capsules, although some samples also arrived in the form of soft gel capsules, gummies, oral strips, and liquid drops

Tablets and capsules: a minimum of 10 capsules or tablets were weighed, opened (capsules) and their contents were mixed in a mortar and pestle. Next, 100 mg of this mixture was weighed and resuspended in 10 mL methanol-water (50:50) *v*/*v*, vortexed briefly for 30 s, and sonicated for 15 min. Next, the mixture was passed through a 0.2 μm polytetrafluoroethylene (PTFE) filter before further analysis.

Gummies or oral strips: an extraction with methanol-water (50:50) *v/v* was performed overnight on a rotary wheel (speed: 300 rpm) in the dark at room temperature. This long incubation was needed to ensure that no visible particles could be observed anymore and to generate optimum recovery. The latter was tested by a fit-for-purpose assay where the amount of melatonin present in a spiked non-melatonin containing gummy (0.1 mg/gummy) was compared to the theoretical amount present in the extract. The assay was performed in triplicate, and the recovery corresponded to 94.2%, indicating acceptable recovery.

Soft gel capsules: The interior of at least 5 capsules was mixed by vortexing, and 100 mg of jelly was weighed into a cylinder and extracted as described above. Similarly to the gummies, a fit-for-purpose assay was performed to test the recovery efficiency for this one sample. Briefly, two sample extraction methods were compared employing a *t*-test. One extraction method consisted of an overnight extraction in 50% methanol (with manual sectioning using a scalpel). For the other sample methodology, the interior contents of soft gel capsules (liquid) were removed by using a syringe and needle. Next, the capsule was rinsed once with a 50% methanol solution and air-dried and weighed. The mass difference between full and empty capsules was used to calculate the mean amount of liquid per capsule. Next, a certain amount of liquid was weighed and extracted as described above, and the amount of melatonin was then calculated based on the amount of liquid present in the soft gel. This test was performed in quadruplicate. Statistical analysis using a *t*-test revealed no significant difference between the melatonin concentrations obtained from the two extraction methods.

Liquid form (spray or drops): Either 10 sprays or 1 mL solution (drops) was diluted 10 times with methanol-water (50:50) *v/v* and filtered. If required, further dilutions were made until the obtained concentration was within the concentration interval for which the appropriate methodology was validated. This interval ranged from 5–250 µg/mL melatonin for the UHPLC-DAD method, while the concentration ranged from 10–200 ng/mL for those samples that required quantification by UHPLC HRAM MS. The measurement uncertainty percentage (termed %MU in the table) was estimated as a confidence interval using the obtained standard deviations of 2 independent preparations injected 3 times.

## Figures and Tables

**Table 1:** The regulation of melatonin in different countries (medicines and/or dietary supplements).

USA	Australia, the UK and Switzerland	Czech Republic, Slovenia and Denmark	Germany	Sweden	Spain and Italy	France	Portugal	**Belgium and The Netherlands**	**Poland**
dietary supplement	Prescription-only, not allowed in dietary supplements	Prolonged-release and immediate-release melatonin are prescription-only medicines. Melatonin is not allowed in dietary supplements	Prolonged-release and immediate-release melatonin are prescription-only medicines. Melatonin is allowed in dietary supplements but lacks clear legislation.	Prolonged-release melatonin is a prescription-only medicine. Immediate-release melatonin medicine is available as over-the-counter (OTC) medicine. Melatonin is not allowed in dietary supplements.	Prolonged-release melatonin is a prescription-only medicine. Immediate-release melatonin medicine is available as over-the-counter (OTC) medicine. Melatonin is allowed in dietary supplements up to a daily dose of 1 mg.	Prolonged-release melatonin is a prescription-only medicine. Melatonin is allowed in dietary supplements provided the daily dose does not exceed 2 mg.	Prolonged-release melatonin is a prescription-only medicine. Immediate-release melatonin medicine is available as over-the-counter (OTC) medicine. Melatonin is allowed in dietary supplements provided the daily dose does not exceed 2 mg.	Prolonged-release melatonin is a prescription-only medicine. Immediate-release melatonin medicine is available as over-the-counter (OTC) medicine. Melatonin is allowed in dietary supplements provided that the daily dose does not exceed 0.3 mg.	Over-the-counter (OTC) medicine, no prescription required Melatonin is allowed in dietary supplements up to a daily dose of 1 mg

**Table 2:** Summary of the UHPLC and LC-MS characteristics and the performance characteristics for the different quantification methodologies.

Method	Molecule	RT (min)	Precursor Ion (*m*/*z*)	Fragment Ions (*m*/*z*) and Their Relative Intensities	SDL	LOQ	Linear Range
LC-DAD	Melatonin	2.5	n.a.	n.a.	1 µg/mL (20 µg/g)	5 µg/mL (100 µg/g)	5–250 µg/mL
LC-MS	Melatonin	2.7	233.128 [M + H]^+^	159.068 (100%) 174.091 (80%) 143.073 (30%) 131.073 (30%)	0.5 ng/mL (10 ng/g)	10 ng/mL (200 ng/g)	10–200 ng/mL
	Melatonin-D3	2.7	236.147 [M + H]^+^				

Abbreviations: RT = retention time; SDL = smallest detection limit; LOQ = limit of quantification; n.a. = not applicable.

**Table 3:** Summary of the validation data of the quantification methodology using UHPLC-DAD or UHPLC-HRAM MS, including trueness, precision, accuracy, and relative expanded uncertainty.

		UHPLC-DAD		UHPLC-HRAM MS	
Linearity expressed as R^2^		/	0.99995	/	0.99999
Trueness	Relative bias (%)	5 µg/mL	1.3	10 ng/mL	1.6
		25 µg/mL	3.5	20 ng/mL	0.9
		50 µg/mL	2.9	60 ng/mL	1.0
		125 µg/mL	1.7	100 ng/mL	0.8
		250 µg/mL	3.2	200 ng/mL	0.4
Precision	Intermediate precision (%)	5 µg/mL	1.7	10 ng/mL	−1.2
		25 µg/mL	1.4	20 ng/mL	−0.4
		50 µg/mL	2.2	60 ng/mL	−0.4
		125 µg/mL	−1.2	100 ng/mL	0.8
		250 µg/mL	−1.5	200 ng/mL	−0.5
	Repeatability (%)	5 µg/mL	2.8	10 ng/mL	1.7
		25 µg/mL	4.0	20 ng/mL	1.6
		50 µg/mL	2.9	60 ng/mL	1.5
		125 µg/mL	2.2	100 ng/mL	1.4
		250 µg/mL	3.4	200 ng/mL	0.8
Accuracy	ß-expectation tolerance limits (%)	5 µg/mL	[−4.9; 2.5]	10 ng/mL	[−9.4; 12.7]
		25 µg/mL	[−4.8; 4.1]	20 ng/mL	[−9.1: 11.8]
		50 µg/mL	[−4.4; 3.6]	60 ng/mL	[−4.9: 9.3]
		125 µg/mL	[−3.1; 4.7]	100 ng/mL	[−7.5: 5.0]
		250 µg/mL	[−3.6;−2.5]	200 ng/mL	[−9.9: 6.9]
Uncertainty	Relative expanded uncertainty (%)	5 µg/mL	3.5	10 ng/mL	6.4
		25 µg/mL	3.5	20 ng/mL	8.6
		50 µg/mL	3.4	60 ng/mL	6.1
		125 µg/mL	3.1	100 ng/mL	4.8
		250 µg/mL	1.9	200 ng/mL	7.2

R^2^ of the linear relationship between the theoretical and measured concentration.

**Table 4:** Information provided on the label compared with the quantity detected of melatonin.

n°	Labeled Health Claim	Lot n° and Expiration Date Present	Galenic Form	Amount per Serving Claimed, mg	Amount per Serving Found, mg (% MU) ^a^	% Label Accuracy
**Registered online pharmacies and brick-and-mortar drug stores**						
L1	No health claim	Yes	Capsules	0.295	0.22 (0.5)	75
L2	Sleep support and general relaxant ^b^	Yes	Capsules	0.29	0.31 (2.0)	105
L3	Sleep support ^b^	Yes	Tablets	0.290	0.26 (4.2)	89
L4	Sleep support ^b^	Yes	Tablets	0.1	0.12 (1.8)	117
L5	No health claim	Yes	Tablets	0.290	0.22 (2.2)	76
L6	Sleep aid	Yes	Tablets	0.295	0.24 (2.1)	83
L7	No health claim labeled	Yes	Tablets	0.1	0.18 (5.2)	182
L8	Sleep support ^b^	Yes	Tablets	0.295	0.11 (1.4)	40
L9	Sleep support ^b^	Yes	Capsules	0.298	0.24 (0.8)	81
L10	Sleep support ^b^	Yes	Capsules	0.295	0.22 (1.5)	76
L11	No health claim	Yes	Tablets	0.145	0.14 (2.3)	97
L12	Sleep support ^b^	Yes	Tablets	0.295	0.35 (1.2)	119
L13	Sleep support and relaxant ^b^	Yes	duocapsule	0.295	0.25 (0.8)	85
L14	Sleep support ^b^	Yes	Tablets	0.29	0.31 (3.0)	107
L15	No health claim	Yes	Tablets	0.29	0.25 (1.6)	85
L16	No health claim	Yes	Tablets	0.299	0.32 (0.3)	107
L17	Sleep support ^b^	Yes	Tablets	0.29	0.21 (0.4)	71
L18	Sleep support	Yes	Tablets	1	0.90 (0.5)	91
L19	Sleep support ^b^	Yes	Oral spray	0.19	0.18 (1.2)	95
L20	Sleep support ^b^	Yes	Oral spray	0.283	0.3 (0.5)	102
L21	Sleep support ^b^	Yes	Gummies ^c^	0.290	0.295 (2.7)	102
L22	Sleep support ^b^	Yes	Gummies ^c^	0.295	0.24 (3.0)	81
L23	Sleep support and relaxant ^b^	Yes	Gummies ^c^	0.295	0.38 (1.8)	129
L24	Sleep support ^b^	Yes	Soft gel	0.299	0.19 (3.0)	74
L25	Sleep support	Yes	Tablet	3	2.5 (1.9)	83
**Rogue online pharmacies and e-commerce sites selling products with a daily melatonin dose > 0.3 mg**						
I1	Healthy sleep cycle	Yes	Capsules	10	10.71 (0.2)	107
I2	Healthy sleep cycle	Yes	Capsules	5	4.52 (0.2)	90
I3	Sleep support	No ^d^	Gummies	5	5.32 (0.1)	106
I4	Promotes restful sleep	Yes	Capsules	1	0.93 (0.7)	93
I5	Sleep support	Yes	Capsules	5	4.18 (0.2)	84
I6	Sleep support	Yes	Capsules	3	3.20 (0.4)	107
I7	Sleep support	Yes	Tablets	3	2.91 (0.6)	97
I8	Sleep support	Yes	Capsules	3	3.76 (0.5)	125
I9	Sleep support	Yes	Capsules	10	10.35 (0.6)	104
I10	Promotes a healthy sleep/wake cycle and may reduce the effects of jet lag	Yes	Capsules	3	3.01 (0.4)	100
I11	Sleep promotion	Yes	Tablets	3	4.09 (0.4)	136
I12	Supports healthy sleep	Yes	Oral drops	3	3.32 (0.2)	111
I13	Supports healthy sleep	Yes	Capsules	1	1.43 (2.5)	143
I14	Supports healthy sleep	Yes	Capsules	2	2.71 (0.1)	136
I15	Supports healthy sleep	Yes	Tablets	5	4.98 (0.1)	100
I16	Supports healthy sleep	Yes	Tablets	10	9.72 (0.3)	97
I17	Promotes a healthy sleep cycle	Yes	Capsules	3	3.88 (0.1)	129
I18	Sleep support	Yes	Capsules	10	17.2 (0.6)	172
I19	Sleep support	Yes	Gummies ^c^	5	4.58 (0.6)	92
I20	Restful sleep	Yes	Capsules	10	11.54 (0.1)	115
I21	Nighttime sleep aid	Yes	Tablets	12	14.36 (0.1)	120
I22	Sleep aid	Yes	Soft gels	10	10.31 (0.3)	103
I23	Promotes a healthy sleep cycle	Yes	Capsules	20	23.43 (0.2)	117
I24	Nighttime sleep aid	Yes	Capsules	10	13.81 (0.5)	138
I25	Restful sleep	Yes	Oral patch	10	8.16 (1.7)	82

^a^ The % MU equals the percentage uncertainty of the measurement, which in turn is expressed as the confidence interval using the standard deviation of the generated quantification results. ^b^ Claim not attributed to melatonin but due to other listed ingredients. ^c^ The container was closed off with a child-proof lid. ^d^ The lot number was present, but the expiration date was missing.

## Data Availability

Data are contained within the article or supplementary material.

## Supplementary Materials

The following supporting information can be downloaded at: https://www.mdpi.com/article/10.3390/molecules30122647/s1, Figure S1: Chromatograms obtained for the different matrices; Table S1: Additional information provided on the label of the different dietary supplements; Table S2: Summary of the obtained recoveries for the UHPLC-DAD analysis of different concentrations in different matrices; Table S3: Summary of the obtained recoveries for the UHPLC-HRAM MS/MS analysis of different concentrations in different matrices.
