# Supplementary material for: Validated UHPLC Methods for Melatonin Quantification Reveal Regulatory Violations in EU Online Dietary Supplements Commerce
Source: Molecules. 2025 Jun 19;30(12):2647. doi: 10.3390/molecules30122647 (PMC12196456; doi:10.3390/molecules30122647)
Supplement: Supplementary file 1 [file molecules-30-02647-s001.zip › molecules-3698655-supplementary.pdf]

Supplemental table S1. Additional information provided on the label of the different dietary supplements .

n°	Country of manufacturing	Country of shipment	Contraindications labeled	Other active substances declared on the label
**Legal supply chain**				
**L1**	Belgium	Belgium	Not suitable for children under the age of 12 or pregnant or lactating women. Keep out of reach and sight of children	-
**L2**	Belgium	Belgium	Not suitable for children under the age of 12. Keep out of reach of children.	Herbal extracts (*Melissa officinalis*, *Valeriana officinalis*, *Eschscholzia californica*, *Passiflora incarnata*), different vitamins, magnesium, and selenium
**L3**	Belgium	Belgium	Not suitable for children. Keep out of reach of young children.	Herbal extract (*Valeriana officinalis*)
**L4**	Belgium	Belgium	Not suitable for children under the age of 12 or pregnant or lactating women. Consult your medical doctor or pharmacist before using this supplement when using medication.	Herbal extract (*Valeriana officinalis* and *Passiflora incarnata*) and magnesium
**L5**	Belgium	Belgium	Not suitable for children under the age of 18 or pregnant or lactating women. Keep out of reach of children.	-
**L6**	Belgium	Belgium	Not suitable for children under the age of 12. Keep out of reach and sight of children.	-
**L7**	The Netherlands	Belgium	Not suitable for children under the age of 12 or pregnant or lactating women. Keep out of reach of children.	-
**L8**	Belgium	Belgium	Not suitable for children. Keep out of reach of young children.	Herbal extract (*Valeriana officinalis, Eschscholzia californica* and *Spirulina platensis*)
**L9**	Belgium	Belgium	Not suitable for children under the age of 12. Pregnant or lactating women should contact a healthcare professional before the consumption of the supplement. Keep out of reach of young children.	Herbal extract (*Valeriana officinalis* and *Humulus lupulus*) and vitamins
**L10**	The Netherlands	Belgium	Not suitable for children under the age of 12 or pregnant or lactating women. Consult your medical doctor or pharmacist before the usage of this supplement when using anti-depressants or other sleep medication.	Herbal extracts (*Melissa officinalis*, *Valeriana officinalis*, *Eschscholzia californica*, *Passiflora incarnata*, and *Humulus lupulus*) and vitamin B12
**L11**	Denmark	Belgium	Not suitable for children under the age of 12 or pregnant or lactating women. Keep out of reach of young children.	Vitamin B3
**L12**	Belgium	Belgium	Not suitable for children under the age of 12. Keep out of reach of young children.	Herbal extract (*Valeriana officinalis*, *Melissa officinalis*, *Passiflora incarnata*, and *Camellia sinensis*) and different vitamins
**L13**	Belgium	Belgium	Not suitable for children under the age of 18. Keep out of reach of children.	Herbal extract (*Passiflora incarnata*), lavender oil and vitamin B6
**L14**	Belgium	Belgium	Not suitable for children, adolescents or pregnant or lactating women. Not suitable for people suffering from inflammation of auto-immune conditions. Consult your medical doctor or pharmacist before the usage of this supplement when suffering from asthma, epileptic seizures, and personality and behavioral disorders. Keep out of reach of children.	Herbal extract (*Passiflora incarnata*, *Eschscholzia californica*), cherry powder, and different vitamins
**L15**	Belgium	Belgium	Keep out of reach of children.	Vitamin B6

**L16**	The Netherlands	Belgium	Not recommended for children under the age of 12 or pregnant or lactating women. Keep out of reach of young children.	-
**L17**	Belgium	Belgium	Not recommended for children under the age of 18 or pregnant or lactating women. Not recommended in case of using antidepressants, alcohol, benzodiazepines, and when suffering from auto-immune conditions. Keep out of reach of young children.	*Eschscholzia californica*, tryptophan, lysine, different vitamin B
**L18**	Italy	Belgium	Keep out of reach of young children under the age of 3.	*Valeriana officinicalis*, *Matricaria chamomilla*, *Lavandula angustifolia*, and vitamin B6
**L19**	Belgium	Belgium	Not suitable for children under the age of 12. Not recommended for pregnant or lactating women without medical advice. Keep out of reach of young children. Excessive use can result in laxative effects.	Extract of *Papaver rhoeas* and *Melissa officinalis*
**L20**	Belgium	Belgium	Not suitable for children under the age of 12. Keep out of reach of young children.	Extracts of *crateagus monogyna and crateagus laevigata*
**L21**	Belgium	Belgium	Not suitable for children under the age of 12. Keep out of reach of children	Herbal extracts (*Matricaria chamomilla*, *Passiflora incarnata*, and *Melissa officinalis)*
**L22**	Belgium	Belgium	Keep out of reach of young children. From the age of 12. Not recommended during pregnancy and breastfeeding. If you're under medical treatment (antidepressant, or another sedative), ask your doctor for advice.	Herbal extracts (*Melissa officinalis, Eschscholzia californica and Tillia cordata), concentrate of Daucus carrotus, Ribes nigrum and vitamin B6*
**L23**	Belgium	Belgium	Not recommended for pregnant or lactating women and children under the age of 12. Keep out of reach of young children.	Herbal extracts (*Valeriana officinalis, Eschscholzia californica, Papaver rhoeas)* and gamma-aminobutyric acid
**L24**	Belgium	Belgium	Not suitable for children under the age of 12. Keep out of reach of children	Extract of *Valeriana officinalis* and hemp seed oil
**L25**	Germany	Belgium	For people 16 years of age or older. Keep out of reach of young children. Use the product carefully when suffering from diabetes.	-
**Suspected illegal supply chain**				
**I1**	USA	The Netherlands	For adults only. Not suitable for pregnant or lactating women. Consult a physician if taking medication or have a medical condition. Keep out of reach of children.	-
**I2**	USA	The Netherlands	For adults only. Not suitable for pregnant or lactating women. Consult a physician if taking medication or have a medical condition. Keep out of reach of children.	-
**I3**	USA	The Netherlands	For adults only. Not suitable for pregnant or lactating women. Consult a physician if taking medication or have a medical condition. Keep out of reach of children.	-
**I4**	USA	The Netherlands	If pregnant or lactating consult a health care professional before using this product. Keep out of reach of children.	Niacin, vitamin B6, L-tryptophan, herbal extract (*Matricaria chamomilla*, *Valeriana officinalis*), and inositol

**I5**	USA	The Netherlands	For adults only. If pregnant, nursing, taking any medications or having any medical condition, consult your doctor before use. Keep out of reach of children.	-
**I6**	USA	The Netherlands	For adults only. If pregnant, nursing, taking any medications or having any medical condition, consult your doctor before use. Keep out of reach of children	5- hydroxytryptophan
**I7**	USA	The Netherlands	For adults only. Not suitable for pregnant or lactating women. Not suitable if suffering from an auto-immune condition or depressive disorder or are under 16 years of age. Keep out of reach of children.	-
**I8**	USA	The Netherlands	For adults only. Not suitable for pregnant or lactating women. Not suitable if suffering from an auto-immune condition or depressive disorder or are under 16 years of age. Keep out of reach of children.	-
**I9**	USA	The Netherlands	For adults only. Not suitable for pregnant or lactating women. Not suitable if suffering from an auto-immune condition or depressive disorder or are under 16 years of age. Keep out of reach of children.	-
**I10**	USA	The Netherlands	Not for use by people under the age of 18 with epilepsy, or by pregnant and breastfeeding women. Not recommended for consumption with blood thinners and alcohol.	-
**I11**	USA	The Netherlands	For occasional short-term use only. Consult a physician before using this product if under the age of 18, taking medication, have any medical condition, are pregnant or lactating, or have an autoimmune condition or depressive disorder.	-
**I12**	USA	Bulgaria	If you are under medical supervision or using any tranquilizers or sedatives, seek the advice of your healthcare professional before using. Consult your physician before using if you have an autoimmune condition, depressive disorder, or are pregnant or lactating. Not for use by children under 12 years of age. Keep out of reach of children.	-
**I13**	USA	Bulgaria	If you are under medical supervision or using any tranquilizers or sedatives, seek the advice of your healthcare professional before using. Consult your physician before using if you have an autoimmune condition,	-

			depressive disorder, or are pregnant or lactating. Not for use by children under 12 years of age. Keep out of reach of children.	
**I14**	USA	Bulgaria	If you are under medical supervision or using any tranquilizers or sedatives, seek the advice of your healthcare professional before using. Consult your physician before using if you have an autoimmune condition, depressive disorder, or are pregnant or lactating. Not for use by children under 12 years of age. Keep out of reach of children.	-
**I15**	USA	Bulgaria	If you are under medical supervision or using any tranquilizers or sedatives, seek the advice of your healthcare professional before using. Consult your physician before using if you have an autoimmune condition, depressive disorder, or are pregnant or lactating. Not for use by children under 12 years of age. Keep out of reach of children.	-
**I16**	USA	Bulgaria	If you are under medical supervision or using any tranquilizers or sedatives, seek the advice of your healthcare professional before using. Consult your physician before using if you have an autoimmune condition, depressive disorder, or are pregnant or lactating. Not for use by children under 12 years of age. Keep out of reach of children.	vitamin B6
**I17**	USA	The Netherland s	For adults only. Not suitable for pregnant or lactating women. Do not use in conjunction with alcoholic beverages. Consult a physician if taking medication or have a medical condition. Keep out of reach of children.	-
**I18**	USA	The Netherland s	For occasional short-term use only. Consult a physician before using this product if under the age of 18, taking medication, have any medical condition, are pregnant or lactating, or have an autoimmune condition or depressive disorder. Keep out of reach of children.	vitamin B6
**I19**	USA	The Netherland s	For adults only. Not suitable for pregnant or lactating women. Not suitable if suffering from an auto-immune condition or depressive disorder or are under 16 years of age. Keep out of reach of children.	-

**I20**	USA	The Netherlands	Not intended for use by pregnant or nursing women. Consult your doctor if you are taking any medication or have any medical condition. Keep out of reach of children.	Theanine, herbal extracts (*Valeriana officinalis*, *Matricaria chamomilla*, and *Passiflora incarnata*), Ashwagandha, tryptophan, and vitamin B6
**I21**	Romania	The Netherlands	Consult your doctor before using when you are suffering from a medical condition.	-
**I22**	Romania	The Netherlands	Consult your doctor before using when you are suffering from a medical condition.	-
**I23**	USA	The Netherlands	For adults only. Not suitable for pregnant or lactating women. Do not use in conjunction with alcoholic beverages. Consult a physician if taking medication or have a medical condition. Keep out of reach of children.	-
**I24**	USA	The Netherlands	Not intended for use by pregnant or nursing women. Consult your doctor if you are taking any medication or have any medical conditions. Keep out of reach of children.	
**I25**	India	The Netherlands	Not intended for use by pregnant or lactating women or anyone undergoing medical treatment. For adult use only. Keep out of reach of children.	*Valeriana officinalis*, herbal extracts (*Matricaria chamomilla and Passiflora incarnata*), vitamin B6, Ashwagandha, and tryptophan

Supplemental Table S2. Summary of the obtained recoveries for the UPHLC-DAD analysis of different concentrations in different matrices.

	µg/mL	Recovery (%)
**Matrix 1**	5	100
	25	104.3
	50	102.3
	125	98.9
	250	103.1
**Matrix 2**	5	98.7
	25	102.7
	50	103.2
	125	**97.6**
	250	99.8
**Matrix 3**	5	**104.8**
	25	99.4
	50	103.5
	125	101.4
	250	102.5

Matrix 1= dried lemon balm; Matrix 2= dietary supplement consisting of different vitamins B and vitamin C; Matrix 3= dried *Valeriana officinalis* powder. The numbers in bold indicate the lowest and highest values obtained for the recovery.

Supplemental Table S3. Summary of the obtained recoveries for the UPHLC-HRAM MS/MS analysis of different concentrations in different matrices.

	ng/mL	Recovery (%)
**Matrix 1**	10	96.5
	20	100.2
	60	97.7
	100	98.7
	200	103.1
**Matrix 2**	10	**92.7**
	20	98.9
	60	105.7
	100	102.3
	200	104.2
**Matrix 3**	10	98.6
	20	106.1
	60	104.0
	100	100.6
	200	**110.3**

Matrix 1= dried *Humulus lupulus*; Matrix 2= dried *Passiflora incarnata*; Matrix 3= dried *Cannabis sativa*. The numbers in bold indicate the lowest and highest values obtained for the recovery.

A

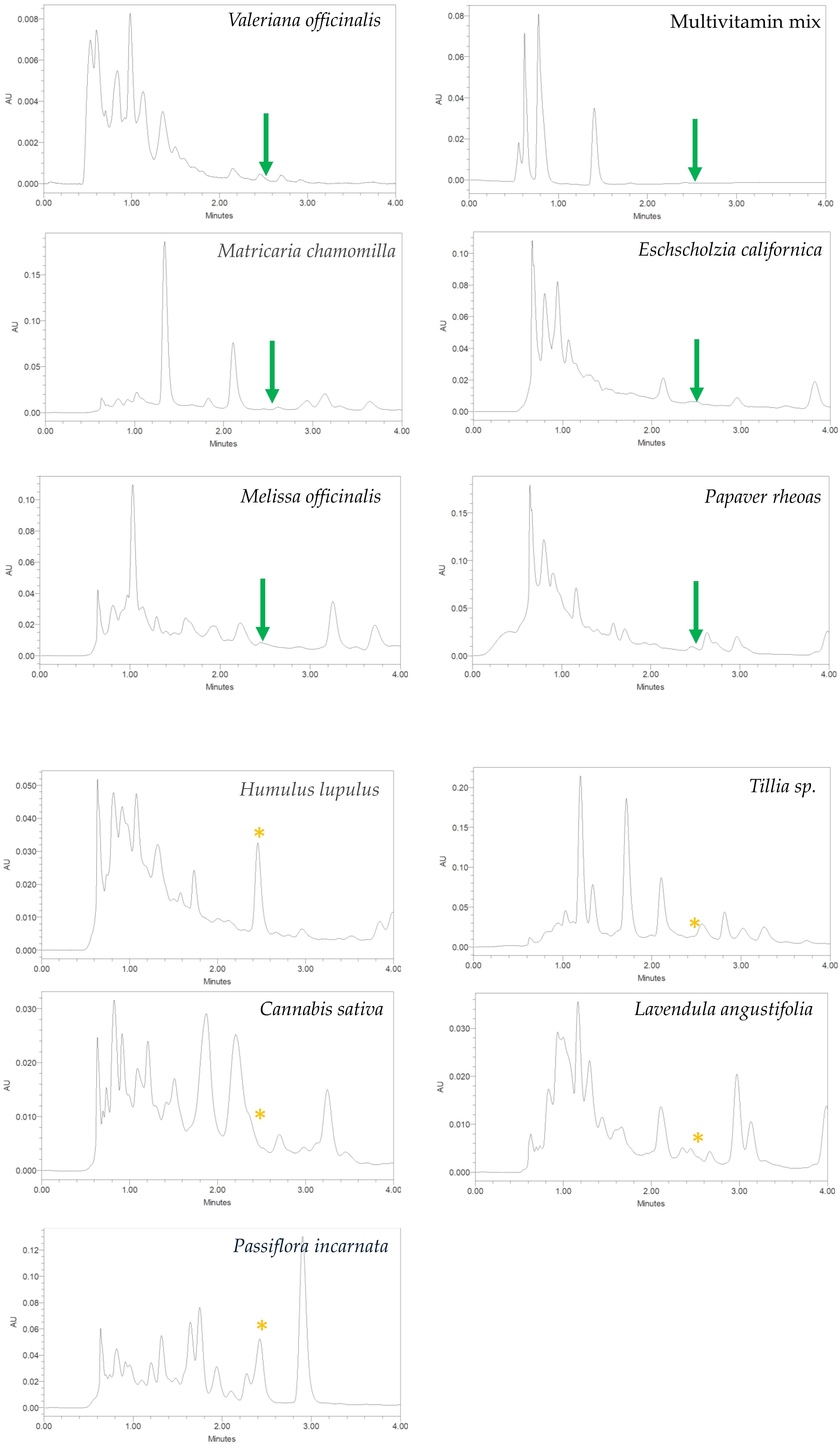

Valeriana officinalis
Multivitamin mix
Matricaria chamomilla
Eschscholzia californica
Melissa officinalis
Papaver rheoas
Humulus lupulus
Tillia sp.
Cannabis sativa
Lavendula angustifolia
Passiflora incarnata
AU
Minutes

B

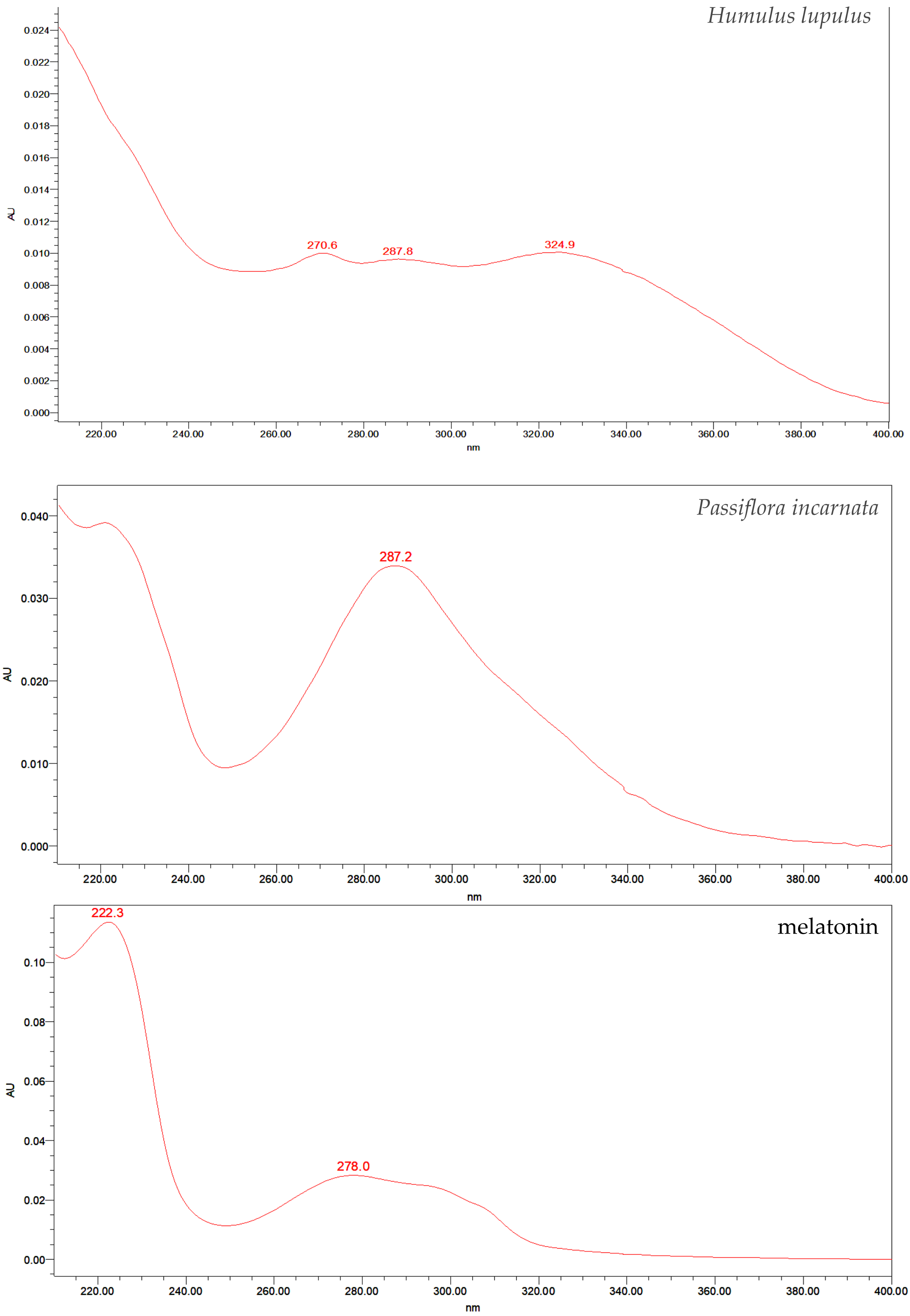

Supplemental Figure S1. Chromotograms obtained for the different matrices (a) and comparison of the UV spectrum of the peak eluting at 2,5 minutes for either hop or passionflower (b)
